# Cochlear inflammaging: cellular and molecular players of the innate and adaptive immune system in age-related hearing loss

**DOI:** 10.3389/fneur.2023.1308823

**Published:** 2023-11-22

**Authors:** Shailee Parekh, Tejbeer Kaur

**Affiliations:** Department of Biomedical Sciences, Creighton University School of Medicine, Omaha, NE, United States

**Keywords:** age-related hearing loss, cochlea, inflammaging, immunosenescence, macrophage, cytokines, complement, inflammasome

## Abstract

Age-related hearing loss is the most common sensory disorder worldwide that contributes to numerous health conditions in the aging population. Despite its prevalence, current treatments, including hearing aids, are unsatisfactory in improving hearing deficits or slowing or reversing its pathophysiology. Immunosenescence is a key driver of neurodegenerative disease, and a similar mechanism has recently come to attention in age-related hearing loss. Imbalanced levels of cytokines and chemokines contribute to aberrant immune cell activity and a chronic pro-inflammatory microenvironment that may lead to degradation of inner ear structure and function. Macrophages, typically guardians of organ homeostasis, are found to develop dysregulated activity with aging due to unidentified factors, and they interact with other components of the innate immune system to damage sensory hair cells, synapses, neurons, and other structures of the inner ear critical to sensory signal transmission. They also increasingly trigger the inflammasome, a protein complex involved in inflammatory cell death, and the complement cascade, to perpetuate a cycle of inflammation and cellular damage in the cochlea, resulting in hearing loss. Senescence in certain T cell populations have indicated a role of adaptive immunity in age-related hearing loss as well. Deciphering the mechanisms of immune dysregulation is a critical first step in producing targeted therapies for hearing loss. This brief review describes the current and emerging research surrounding the dysregulation of the innate and adaptive immune systems in age-related hearing loss and its parallels with other neurodegenerative diseases.

## 1 Introduction

Age-related hearing loss (ARHL) is one of the most common health disorders in the aging population, affecting over a third of adults over age 65 (1). It is characterized by progressive difficulty detecting high frequency sounds and impaired ability to understand speech, especially amidst background noise, a phenomenon known as hidden hearing loss (1). Although ARHL was previously considered a harmless by-product of aging, it has been linked with significant negative physical and mental health outcomes including anxiety, depression (2), dementia (3), reduced mobility, and falls (4). Despite its prevalence and major impact on quality of life, ARHL currently has no molecular treatments or preventative therapies, and its complex pathobiology is still under investigation.

Recently, dysregulation of the immune system has come into light as a major pathological driver of ARHL. The term “inflammaging” describes the low-grade, sterile, chronic inflammatory state in the body’s tissues with age. Chronic inflammation plays a role in multiple systemic diseases such as diabetes, as well as neurodegenerative diseases, including Alzheimer’s disease (AD), Parkinson’s disease (PD), multiple sclerosis, and retinal degeneration (5). During aging, immune cells undergo senescence, a process in which they lose the ability both to mount the normal immune response and to resolve inflammation after acute insults. Increased systemic levels of inflammatory markers, including C-reactive protein, interleukin-6 (IL-6), and white blood cell counts, are observed in neurodegenerative processes and ARHL, suggesting that inflammation is a hallmark of the aging brain and inner ear (5–8). Newer studies are exploring the role of gut microbiota in inflammaging, noting how pro-inflammatory diets such as foods high in sugar correlate with systemic inflammatory markers and severity of hearing loss (9, 10). Conversely, long-term exercise is thought to delay progression of neurodegenerative processes and ARHL through the dampening of inflammation (11, 12). Inflammaging can impair neural and sensory networks through several interrelated processes, many of which have been well-characterized in neurodegenerative diseases. Similar mechanisms are now being elucidated in ARHL as well.

**Figure 1:**
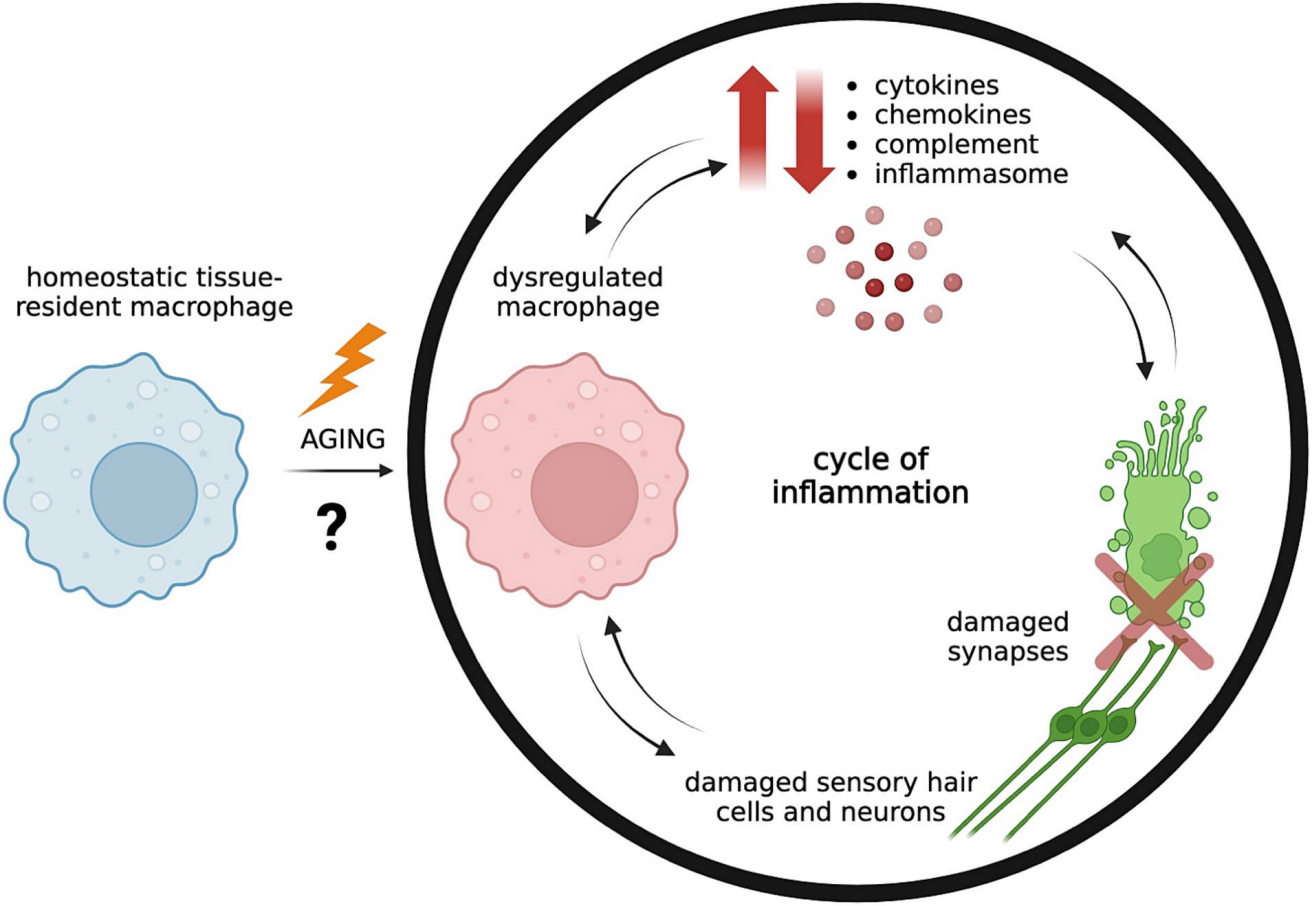
Aging is associated with dysregulated tissue-resident macrophages in the cochlea due to unknown driving factors, leading to imbalanced levels of pro-inflammatory and pro-resolving mediators. Dysregulated macrophages may release uncontrolled pro-inflammatory cytokines and chemokines and trigger activation of the complement system and inflammasome pathway. Inflammaging contributes to weakening of the blood-labyrinth barrier of the cochlea which allows further infiltration of inflammatory mediators from the circulation into normally tightly controlled environments, resulting in damage of sensory hair cells and neurons and their synaptic connections (5, 13). Continued damage of neurons and synapses increasingly trigger macrophage activation, which contributes to a cycle of unresolved inflammation and tissue destruction (14). Created with BioRender.com.

This brief review summarizes current research on the cellular and molecular components of the innate immune system in ARHL, including the role of inflammatory cytokines, chemokines, inflammasomes, the classical complement pathway, and macrophages, as well as the interactions of each of these players with inner ear sensory structures (Figure 1). It also discusses emerging research of the involvement of the adaptive immune system in ARHL which thus far has largely been overlooked. A better understanding of immunosenescence in ARHL can elucidate future therapeutic avenues for a very prevalent and debilitating condition that currently has no preventative medicines or molecular treatments that target its underlying pathology.

## 2 Cellular and molecular players of the innate immune system in ARHL and neurodegenerative disease

### 2.1 Inflammatory cytokines

In addition to increased systemic inflammatory markers, neurodegenerative diseases and ARHL display increased localized inflammation within the brain and cochlea (13, 15, 16). Cytokines are proteins that modulate the immune response and are central to immune cell differentiation, migration, phenotype, and function. Emerging research on the mechanisms of pro-inflammatory cytokines in ARHL suggest that their pathological effects occur when their levels become imbalanced (16).

#### 2.1.1 Interleukin-1β

Interleukin-1β (IL-1β) is a major pro-inflammatory cytokine that demonstrates a complex role in AD pathology (16). It is released by microglia (brain macrophages, a type of innate immune cell) through the inflammasome pathway (17). While it has been found to diminish long-term potentiation (LTP) and memory consolidation in AD mice models through increased tau phosphorylation and disruption of the blood–brain barrier, it also facilitates the degradation of amyloid beta (Aβ) plaques, a major pathological driver in AD, thereby demonstrating both damaging and protective effects (18). In mice studies of ARHL, increased IL-1β is associated with synapse loss and worsening of hearing thresholds (19). Further, genetic polymorphisms in the IL-1 receptor in humans have been found to influence individual susceptibility to sudden sensorineural hearing loss (20). This indicates that IL-1β activity plays a role in driving hearing loss, but its specific interactions with inner ear components during aging are yet to be characterized.

#### 2.1.2 Tumor necrosis factor α

Tumor necrosis factor α (TNF-α), a powerful inflammatory cytokine central to the immune response, triggers multiple cell signaling pathways involved in cell death, proliferation, and differentiation. It is produced by microglia in the central nervous system (CNS) and macrophages in the ear and works through two different receptors, TNFR1 and TNFR2, to produce both neurotoxic and neuroprotective effects (21). For instance, necroptosis, a form of inflammatory cell death, is dependent on TNF signaling in AD mice models (22). TNF-α also impedes the electron transport chain in mitochondria and results in increased production of reactive oxygen species (ROS) in the aging ear which are toxic to neurons (23). On the other hand, TNF-α is important for the maintenance and regeneration of myelin, and TNF blockers are known to exacerbate symptoms of multiple sclerosis, an autoimmune disease characterized by inflammatory injury to myelin in the CNS (21). TNF-α deficient mice have very early hearing loss, especially at high frequencies, and loss of stereocilia in the outer hair cells (24). It is likely the loss of balance of TNF levels with aging that predisposes the inner ear to both inflammatory damage and destructive cell signaling pathways. While low concentrations of TNF-α facilitate hair cell survival through activation of the nuclear factor-κB (NF-κB) transcription factor, high concentrations lead to hair cell apoptosis through initiation of the Caspase-3 cascade (25).

#### 2.1.3 Macrophage migration inhibitory factor

Macrophage migration inhibitory factor (MIF) is a pro-inflammatory cytokine with strong expression in the nervous system and is involved in a multitude of processes, including initiation of the inflammatory response and release of other cytokines, as well as prevention of apoptosis and ROS production (26, 27). Zhang et al. found that MIF expression was increased in AD mice models and MIF could directly bind to Aβ plaques (27). MIF deficiency resulted in memory impairment on the Morris water maze whereas overexpression of MIF preserved neural cells from Aβ toxicity (27). On the other hand, Nasiri et al. demonstrated that MIF inhibition preserved contextual memory in mice with Streptozotocin-induced hippocampal impairment, and MIF was associated with hyperphosphorylated tau levels (26). Furthermore, increases in IL-1β and IL-6, potent proinflammatory cytokines, in response to Streptozotocin were MIF dependent (26). Conflicting evidence as to whether MIF is protective or pathogenic in AD is likely due to its ability to activate multiple intracellular signaling cascades. Additionally, MIF may promote neural survival in early stages of Aβ plaque deposition but result in excessive inflammatory damage at later stages that accelerates AD pathology (27).

MIF is also expressed in the inner ear and early studies suggest that its endogenous downregulation with aging contributes to ARHL (28). MIF knockout mice have significantly increased auditory brainstem response (ABR) thresholds starting at 9 months and display loss of outer hair cells and spiral ganglion neurons (SGNs) (28). MIF is also central to the viability and survival of perivascular-resident macrophage-like melanocytes (PVM/Ms) in the lateral wall during aging (29). PVMs are part of the blood–brain barrier and blood-labyrinth barrier and preserve the integrity of the stria vascularis in the cochlea of the inner ear which is crucial to the development and maintenance of the endocochlear potential, an electrochemical gradient that allows transmission of signals between motile sensory hair cells and corresponding SGNs (29).

### 2.2 Chemokines

Chemokines, cytokines that influence leukocyte migration and infiltration into tissues, are also central players in neurodegenerative processes. CC-chemokine ligand 2 (CCL2), also known as monocyte attractant protein-1, recruits microglia to areas of inflammation and is related to progression of AD, PD, and multiple systems atrophy (16). Its gene expression is upregulated in the mouse cochlea during normal aging (15).

CX_3_CL1, also known as fractalkine (FKN), is constitutively expressed by CNS neurons, SGNs, sensory inner hair cells, and certain types of supporting cells (30). FKN acts on its sole receptor, CX_3_CR1, expressed by macrophages, and thereby regulates macrophage migration and activity. Intact FKN signaling has been shown to play a neuroprotective, anti-inflammatory role in neurodegenerative diseases and to promote neuron and hair cell survival and restoration of inner hair cell ribbon synapses following noise-and ototoxic drug-induced cochlear injury in mice (30–33). Early studies conducted by Hirose et al. demonstrated that bone marrow-derived macrophages are recruited to the mouse inner ear following noise and drug-induced injury to play a phagocytic role (34, 35). When CX_3_CR1 was knocked out, there was an increased inflammatory response in the inner ear with worsening hearing loss. Using a bone marrow chimera in which the bone marrow of wild type mice was irradiated and subsequently transplanted with CX_3_CR1 wild type or CX_3_CR1 knockout marrow, Hirose and colleagues found a positive correlation between levels of recruited macrophages and hair cell damage solely in the CX_3_CR1 knockout group. Together, these studies suggest that CX_3_CR1 is an important protective molecule present on macrophages that regulates their numbers and responses and influences sensory cell survival in the damaged cochlea (35).

Further investigation of the endogenous levels and precise function of FKN and its receptor in the aging cochlea could highlight potential avenues to modulate macrophage numbers and dysregulation in ARHL. In humans, there are two single nucleotide polymorphisms of CX_3_CR1, present in 20–30% of the population, which lead to defective FKN binding and signaling. These polymorphisms have thus far been studied in the pathogenesis and susceptibility of developing multiple sclerosis, age-related macular degeneration, and diabetic retinopathy (30, 36, 37). Further study on how these polymorphisms are associated with the vulnerability of individuals to develop sensorineural hearing loss will provide valuable information about the role of FKN signaling in ARHL.

### 2.3 Inflammasome

Inflammasomes are intracellular oligomeric protein complexes that are involved in innate immune defense through fast, inflammatory cell death called pyroptosis (38). Inflammasomes are expressed by several cell types, including macrophages and microglia, and consist of three components: a sensor molecule, an adaptor protein which promotes oligomerization, and an effector known as caspase-1 (39). Activation of caspase-1 leads to pyroptosis, leakage of cellular contents, and cleavage and release of pro-inflammatory cytokines IL-1ꞵ and IL-18 (39). The nucleotide-binding domain, leucine-rich-containing family, pyrin domain-containing-3 (NLRP3) inflammasome, classified by its specific sensor molecule, has been indicated in the pathogenesis of many inflammatory disorders including neurodegenerative disease (17). While the inflammasome is critical to defense against damaged proteins, overactivation leads to an uncontrolled inflammatory microenvironment and positive feedback loop, wherein inflammatory cytokines chronically activate microglia and result in microglial dysfunction and neuronal damage.

The NLRP3 inflammasome is triggered by a multitude of factors known as pathogen associated molecular patterns and damage associated molecular patterns (40). These triggers include ROS, ion fluctuations, and lysosomal disruption, all of which are observed to a greater extent in aging tissue (39). Additionally, abnormal protein accumulation such as Aβ and hyperphosphorylated tau in AD, alpha synuclein in PD, and misfolded protein in prion disease increase NLRP3 activation through different mechanisms (17). For example, in PD, alpha synuclein interacts with toll-like receptors (TLR) on microglia to activate the transcription factor NF-κB which upregulates production of NLRP3, pro-IL-1ꞵ, and pro-IL-18 (38). The inflammasome is then activated through a variety of stimuli, including lysosome damage due to microglial engulfment of alpha synuclein aggregates (38). Indeed, PD patients exhibit higher levels of IL-1β and TNF-α in the striatum, as well as increased NLRP3 protein, caspase-1, and IL-1β in peripheral blood mononuclear cells (17).

Not only is NLRP3 inflammasome overactivity observed in neurodegeneration, but NLRP3 may be a key driver of disease. Heneka et al. knocked out NLRP3 in an AD mouse model and found lower levels of Aβ aggregates, protected cognitive function as measured by spatial memory and locomotor activity, and preserved hippocampal synaptic transmission measured by LTP (41). They further suggested that inhibition of the inflammasome could be a potential therapy for AD (41).

In mouse models of ARHL, mRNA and protein studies have shown elevated levels of ROS, NLRP3, caspase-1, IL-1β, and IL-18 in the aging cochlea, primarily in the spiral ligament, spiral limbus, and organ of Corti (19, 42). These findings are accompanied by increased inflammatory markers, IL-6 and TNF-α, and deteriorating hair cells and SGNs in aging ears (19). Uraguchi et al. additionally found elevated mRNA levels of TLR-9, Clec4e, and Card9 in the cochlea of aging mice, which mediate the NLRP3 inflammasome (42). TLR-9 promotes activation of NF-κB which upregulates the expression of multiple inflammatory cytokines, including IL-6 and TNF-α, and primes NLRP3 activation through increased transcription of inflammasome components (43). Clec4e is a member of the C-type lectins family, which are a type of pattern recognition molecules that recognize inflammasome activating stimuli (17). CARD9 is one component of the adaptor protein portion of the inflammasome and aids in oligomerization during inflammasome activation. Interestingly, CARD9 suppresses NLRP3 activation in multiple infectious processes, although its precise activity in the aging central and peripheral nervous system is complex (44).

A recent *in vitro* study looked at the effect of aging and inflammation on ribbon synapses, which are thought to be one the first structures to degenerate in ARHL and to contribute to hidden hearing loss, the inability to distinguish sounds in a noisy background, even in the absence of increased hearing thresholds (1). Feng et al. simulated noise-related and age-related toxicity in the basilar membrane of C57BL/6 J mice (mouse model exhibiting early onset ARHL) cochlea using NMDA + kainate (to simulate glutamate excitotoxicity that is observed in noise-induced injury) and D-galactose (which induces oxidative stress and cell senescence) mediums, respectively (45). There were significantly reduced numbers of ribbon synapses and auditory nerve fibers in both treatment groups compared to control, as well as reduced ribbon synapses in the aging compared to noise toxicity group although statistical analysis was not performed in this case (45). Feng also found increased protein levels of NLRP3, IL-1β, IL-18, caspase-1, and TNF-α in treatment groups compared to control. This suggests that inflammasome activation and resulting inflammation is involved in synaptic damage. However, including an additional subset of NLRP3 deficient mice in the study would have provided more substantial evidence that synapse loss is mediated by inflammasome activity. Additionally, while the NLRP3 inflammasome may be a shared pathological mechanism in both noise-induced hearing loss (46) and ARHL, it is worthwhile to further study the differences between acute vs. chronic inflammasome activation. Perhaps, variation in inflammasome activity can explain the increased synaptic loss that was found in ARHL compared to noise-induced hearing loss models.

### 2.4 Complement cascade

The complement cascade is another component of innate immunity whose aberrant activation has been implicated in a number of age-related neurodegenerative processes, in particular, age-related macular degeneration (AMD) (47). The three complement pathways, the classical, leptin, and alternative pathways, are each initiated by different molecules but converge to result in a protein activation cascade that produces an important defense against injury and infection. This response includes the release of anaphylatoxins, opsonization of pathogens, and formation of the membrane attack complex which perforates target cells (47).

C1q, the initiating molecule of the classical complement pathway, is one of the major complement proteins shown to contribute to the chronic inflammatory response in AMD (47). Dry AMD is a progressive retinal disease that results in loss of central vision and in later stages is characterized by geographic atrophy, caused by the loss of photoreceptors and retinal pigment epithelial cells in the outer layers of the retina (48). Lipid and protein aggregates known as drusen build up under the retinal pigment epithelium (RPE) in geographic atrophy (48). Several components of drusen are known to activate C1q, including C-reactive protein, phosphatidylserine, and serum amyloid protein (47). Additionally, persistent inflammation and damaged RPE results in disruption of the blood-retinal barrier which allows additional complement proteins and C1q-expressing macrophages to enter the outer retina from the circulation (47).

The complement cascade also plays a role in early microglial-mediated synapse elimination and cognitive decline in AD (49–51). While complement mediated synaptic pruning is a normal process in both development and plasticity, it may be aberrantly overactivated in neurodegenerative disease like AD and multiple sclerosis (50). Hong et al. demonstrated that inhibition of C1q, C3, or CR3 (microglial complement receptor) in young, pre-plaque AD mice models prevented microglial engulfment of hippocampal synapses in response to soluble Aβ (52). In aged, plaque-rich AD mice, C3 deficient mice had relatively preserved cognition and synapses despite higher Aβ plaque load (51). This suggests that ongoing complement activation is a necessary component in the neurodegenerative changes that lead to cognitive decline in AD, and that these changes can occur even prior to the appearance of Aβ plaques.

While currently there is limited investigation into complement activity in the inner ear, recent studies do implicate a similar process occurring in ARHL. Seicol et al. labeled C1q in the anteroventral cochlear nucleus (CN) of young, middle-aged, and aged CBA/CaJ mice (mouse model with human-like late onset ARHL) (14). They demonstrated significantly increased intracellular C1q in activated microglia in the CN with age, as well as increased tissue deposition of C1q in the CN, indicating that complement is involved in the post-cochlear signal transmission in ARHL (14). In another study, transcriptomic analysis of differentially expressed genes in the cochlea of young and aged C57BL/6 mice revealed that inflammatory genes were particularly upregulated in aged mice (15). Notably, these included genes for complement proteins C1Qa, C1Qa, C1Qc, C1ra, which are unique to the classical pathway, as well as C4b and C3 (15). Brown et al. additionally highlighted compliment factor B, an activator molecule of the alternative complement pathway, as crucial to the maintenance of auditory function in mice (53). Knocking out complement factor B resulted in sensorineural hearing loss, with increased ABR thresholds and damage to the stria vascularis and auditory nerve myelin sheaths (53). Based on these findings, investigating how the expression of compliment factor B changes with normal aging and whether it plays any significant role in ARHL is another exciting avenue for research.

### 2.5 Tissue-resident macrophages

Microglia, the resident macrophages of the CNS, are essential in host defense and tissue homeostasis. When describing neurodegenerative processes, microglia have often been divided into an inflammatory, cytotoxic M1 phenotype and an anti-inflammatory, neuroprotective M2 phenotype although microglial activity exists along a continuum between these states (54). Microglial responses are activated after neuronal damage through factors such as the release of ATP and changes in ion concentrations (54). Age-related changes in microglia themselves also shift them toward a more reactive phenotype. These include changes in transcriptional and epigenetic factors to favor the release of inflammatory cytokines, increased expression of damage associated molecular pattern receptors on the cell surface, and impaired phagocytic activity (54). The cycle of neural damage and immunosenescence contributes to a persistent dysregulation of microglial activity that is observed in AD, PD, multiple sclerosis, and amyotrophic lateral sclerosis (54). Microglial dysfunction is associated with the loss of dopaminergic neurons in the substantia nigra in PD, cytokine-mediated glutamate toxicity, and deficits in LTP and memory consolidation in AD (54).

Macrophages are found in the inner ear under steady state conditions, playing an analogous role to microglia in the CNS. They are present in areas including the osseous spiral lamina (OSL), basilar membrane, stria vascularis, and spiral ganglion and display heterogeneous morphology throughout the lifespan (55). It is known that macrophages migrate to areas of damage in the mouse inner ear and are vital to debris clearance, synapse recovery, and SGN survival following acute injury (31, 32, 56, 57). Several important age-related changes have been observed as well in cochlear macrophage morphology, migration, and number (58). In mice studies of ARHL, activated macrophages are found in areas of active hair cell degeneration, visualized as large ameboid cells containing multiple vacuoles and grainy cytoplasm and reduced number of cellular processes (55, 58). On the basilar membrane, these active macrophages are found starting at the basal end, corresponding to the area that detects high pitched sounds, and where hair cells and SGNs first start to degenerate in ARHL (55). In the OSL, there is an overall steady increase during aging of CD68, a lysosomal marker of phagocytic activation, found on both resident macrophages and other immune cells (14). Additionally, the macrophage number and area increase in the OSL with age and correlate with the degree of hearing loss (14). In a recent paper, Lang et al. described the interaction between inflammaging, macrophage dysfunction, and strial degeneration in aging CBA/CaJ mice (59). The role of maintaining the endocochlear potential makes the stria vascularis a highly metabolically active, but also vulnerable, epithelium on the lateral wall of the cochlea. Aging mice displayed diminished endocochlear potentials, loss of strial capillary density, and enhanced gene expression of inflammaging-related biological processes starting as early as middle age. Histochemical analysis revealed that the degenerating stria was associated with decreased macrophage-capillary interactions and changes in macrophage morphology into an active ameboid shape. Importantly, gene expression analysis suggested that these macrophages were dysregulated, dysfunctional, and pro-inflammatory (59).

Macrophage distribution and morphology in the human inner ear under steady state conditions is difficult to assess as most research designs rely on the collection of human temporal bones after death. Liu et al. analyzed freshly fixed cochlea isolated from individuals undergoing surgery for petroclival meningioma, which uniquely provided samples that were relatively free of age-related pathologies and artifacts caused by post-mortem specimen collection (60). Similar to mice studies, Liu et al. found macrophages widely distributed in the inner ear displaying a range of different morphologies. Of note, macrophages were found near blood vessels in the stria vascularis, suggesting a role in the maintenance and function of the blood-labyrinth barrier. They were additionally seen in close association with SGNs and were observed near damaged hair cells (60). Lang et al. investigated age-related changes in the human cochlea and found increased levels of macrophage activation and decreased interaction between macrophages and the strial vasculature in older donors compared to younger donors (59). A different study using human temporal bones discovered that macrophages increasingly surround the cochlear nerve myelin sheath in older individuals, suggesting atypical macrophage-glial interactions with aging as well (58). Importantly, the changes in macrophage activation and morphology in these studies are detected even before hair cell degeneration, indicating that macrophage activation is not solely a response to cell death. Rather, macrophage activity may be triggered by changes in neural physiology (58) and may be a driving factor of cell degeneration through release of more inflammatory signals (14, 55). Reviewing studies in both animal models and humans and deciphering the precise role of macrophages in cell degeneration or survival and in ARHL using novel depletion strategies will be necessary for a better understanding.

## 3 The adaptive immune system in ARHL

While ARHL research has largely focused on the innate immune system as a driver of inflammaging, the adaptive immune system also undergoes senescence and is involved in brain pathologies (61). Emerging research on the adaptive immune system in ARHL focuses on T cells, of which there are three main types: CD4+ helper T cells, CD8+ cytotoxic T cells, and regulatory T cells (Treg). During aging, naturally occurring Tregs from the thymus and another type of T cell, IL-1R+ CD4+ T cells, accumulate and accelerate senescence-associated inflammation and neurodegeneration (61). In order to study these two T cell populations in ARHL, Iwai & Inaba used a senescence associated mouse model that experiences accelerated thymic involution, leading to decreased output of properly functioning naive T cells and dysfunctional cellular immunity. Levels of Treg and IL-1R2+ CD4+ T cells increased with age; however, these populations decreased in number after a fetal thymus graft (61). Thymus graft after the onset of ARHL in 12-month-old mice also restored T cell function, SGN density, and hearing function, with levels comparable to young 2-month-old mice (61). In a follow up experiment, they proposed that while lymphocytes did not infiltrate the cochlea, SGN degeneration and blood-labyrinth barrier breakdown was likely facilitated through nitrous oxide production by these immune senescent cells (62). Further, inoculation with CD4+ T cells lacking IL-1R2 inhibited SGN loss (62). IL-1R2 prevents IL-1 signaling in the brain among immune cells, neurons, and glia by acting as a decoy receptor. It binds to IL-1 without any resulting signal transduction (61). While IL-1 was previously discussed to have potential neurodegenerative effects in AD and ARHL, controlled IL-1 signaling is still necessary in microglial function and homeostasis in the neural environment. Further studies should investigate other populations of T cells and additional components of adaptive immunity, such as B cells and immunoglobulins, along with their interactions with the innate immune system in aging and ARHL (63).

## 4 Perspectives on potential therapies for cochlear inflammaging

As inflammaging is a systemic process, methods employed to reduce systemic inflammation have provided some benefit in slowing both age-related neurodegenerative processes and hearing loss (9, 10, 12). For example, long-term exercise in mice was shown to lower inflammatory markers such as CCL2 and TNF-α, and these mice had lower hearing thresholds, preserved strial vasculature, and less hair cell and SGN loss compared to mice that did not exercise (11). Similar impacts of exercise are seen in humans, as there is an association between exercise and milder hearing loss in older adults (64). However, while reduction in systemic inflammation may be a long-term preventative therapy for ARHL, more targeted treatments that directly alter the microenvironment of the inner ear will be more effective to slow, or even reverse, the damage caused by inflammatory mediators in ARHL. One avenue for potential ARHL treatment is through alteration of age-related changes of the complement cascade in the inner ear. Complement inhibitors, particularly C3 and C5 inhibitors, have shown to be effective in slowing geographic atrophy in dry AMD, and numerous clinical trials are ongoing (65, 66). The role of complement factors in ARHL still needs to be characterized further before treatments can be developed. Thus far, C1q and the classical pathway have been identified as potential drivers of disease. Another intriguing route is to investigate the chemokine FKN in ARHL. FKN may protect against glutamate excitotoxicity in the brain and reduce aberrant inflammatory signaling by microglia in neurodegenerative diseases including AD, PD, and diabetic retinopathy (30). FKN’s role in regulating macrophages and protecting the inner ear after noise-and drug-induced injury could indicate that it has a similar function during the aging process. It would be valuable to see how levels of FKN and its receptor change with aging in the cochlea, and perhaps, if reduced endogenous levels of FKN contribute to macrophage dysfunction in the aging ear. In that case, restoration of FKN could potentially be a powerful localized therapy, especially since aberrant macrophage activity is a key player in ARHL pathology. Macrophage activity can also be altered through the replacement of dysregulated, senescent macrophages with new cells. This has been demonstrated in the brain through temporary inhibition of colony-stimulating factor 1 receptor, which repopulates the brain with new microglia. These cells exhibited beneficial, reduced inflammatory activity in response to injury following intracranial hemorrhage (67). Another strategy that has been tested in the brain is the introduction of young blood into aged mice through heterochronic parabionts. These mice had improvement in age-related cognitive deficits as well as synaptic plasticity in the hippocampus (68). Both the removal of senescent cells and pro-inflammatory factors from the blood, such as unbalanced cytokine levels, damage-associated molecular patterns, complement proteins, as well as introduction of cells with anti-inflammatory phenotypes likely contribute to these changes. In the context of ARHL, any treatment will require investigation to determine how best to access the inner ear compartment in order to gain optimal therapeutic efficacy (Table 1).

**Table 1:** Summary of cellular and molecular players of the innate and adaptive immune systems in age-related hearing loss.

	Age-related changes	Studies in the aging inner ear?
Cellular players		
Macrophages	Macrophages accumulate in areas of damage and exhibit a dysregulated phenotype with aging in the inner ear. They have increased phagocytic activity and impaired interactions with the strial vasculature and cochlear nerve. Dysfunctional, senescent macrophages may be a key driver in age-related hearing loss.	Mice (14, 55, 59) Humans (58–60)
T-cells	Senescent Treg and IL-1R2+ CD4+ T cells accumulate with age and accelerate inflammation and loss of spiral ganglion neurons and hearing function in mice.	Mice (61, 62)
Molecular players		
Interleukin-1β	IL-1β is released by the inflammasome pathway and is associated with synapse loss and increased hearing thresholds in aging mice.	Mice (19)
Tumor necrosis factor α	Low concentrations of TNF-α are needed for hair cell survival, and lack of TNF-α leads to early hearing loss in mice. At the same time, high concentrations cause hair cell apoptosis and inflammatory damage. TNF-α levels are dysregulated with aging.	Mice (24, 25)
Macrophage migration inhibitory factor	Endogenous MIF levels decrease with age. Loss of MIF is associated with damaged inner ear components and impaired hearing function in mice. MIF is important to the integrity of the stria vascularis.	Mice (28, 29)
Fractalkine	Fractalkine promotes cell survival in the mouse cochlea following noise-and drug-related injury through regulation of macrophage migration and activity. Studies investigating its role in the aging ear are still needed.	Drug-and noise-induced hearing loss in mice (31, 32, 35)
Inflammasome	Protein and mRNA levels of inflammasome components are upregulated in the aging mice cochlea.	Mice (19, 42, 45)
Complement Cascade	C1q, part of the classical complement pathway, is found in increased levels with age in activated microglia in the cochlear nucleus. Several other complement proteins are upregulated in the aging mouse cochlea.	Mice (14, 15, 53)

## 5 Concluding remarks

With a rapidly aging population, there is an increased need to understand the mechanisms that drive ARHL to develop targeted molecular therapies to slow or reverse its progression. Recently, studies have discovered exciting evidence of innate and adaptive immune system dysfunction in the aging cochlea though current research of its role in ARHL is still precursory. Striking parallels are observed between immunosenescence in ARHL and other age-related CNS disorders. There are many potential avenues to target mechanisms of inflammaging, from modulation of the complement system to restoration of fractalkine or healthy macrophages and T cell populations. Further research is required to explore the changes in the molecular states of immune cells as a function of aging and define the delicate balance between beneficial and destructive inflammation in the inner ear.

## 6 Outstanding questions

What intrinsic and extrinsic factors drive dysregulation and senescence of macrophages and T-cells?

What are the dysregulated macrophage populations in aging ears? Are there different subtypes of dysregulated macrophages?

How can dysregulated macrophages be identified and differentiated from homeostatic tissue-resident macrophages? Are there key markers that can identify and differentiate dysregulated macrophages and T-cells in the cochlea?

How do dysregulated macrophages directly damage sensory structures in the cochlea?

Are there other types of innate and adaptive immune cells that contribute to ARHL?

## Author contributions

SP: Writing – original draft. TK: Writing – review & editing.
